# ER: YAG LASER THERAPY FOR STEATOCYSTOMA MULTIPLEX

**DOI:** 10.4103/0019-5154.70690

**Published:** 2010

**Authors:** Ceyda Tanzer Mumcuoğlu, Mehmet Salih Gurel, Ummuhan Kiremitci, Asli Vefa Turgut Erdemir, Yeliz Karakoca, Osman Huten

**Affiliations:** *From the Department of Dermatology, Istanbul Training and Research Hospital, Turkey. E-mail: ceydatanzer@gmail.com*

Sir,

Steatocystoma multiplex (SM) is characterized by multiple, epithelium-lined, sebum-filled dermal cysts with characteristic sebaceous glands in the cyst wall. SM lesions present with asymptomatic, yellow or skin-colored dermal papules or cysts located most commonly on the trunk, upper arms, scrotum, or chest. Oily material is expressed when incised.[1] Males and females are equally affected and cysts appear during adolescence and early adulthood.[2]

**Figure 1:**
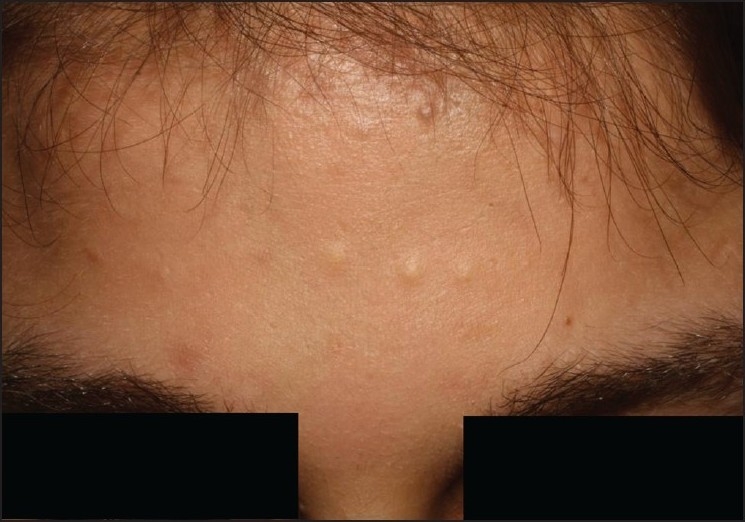
Multiple flesh-coloured to yellow papules, varying in size between 2 and 4 mm on forehead

A 16 year-old female patient presented with a two-year history of skin cysts on the anterior chest, forehead, axillae, and around the knees, follicular hyperkeratosis, bilateral plantar keratoderma, subungual hyperkeratosis, erythematous scaly patches on the elbows and knees, as well as yellow-brown discoloration in all the nails. She had a history of two natal teeth at birth. Physical examination revealed multiple flesh-colored to yellow papules, varying in size between 2 and 4 mm on the anterior chest, forehead, axilla, and neck [Figure 1]. Systemic and laboratory examinations revealed no pathological findings except anemia.

Biopsy specimens were taken from the left anterior chest. Histological examination demonstrated a cystic lesion located in the mid-dermis. The cyst wall was lined by stratified squamous epithelium containing keratinous material; adnexal structures were found. The patient was diagnosed with SM based on the histological and clinical findings. A diagnosis of pachyonychia congenita type 2 was confirmed by the findings of SM, natal teeth, subungual hyperkeratosis, psoriatic patches, keratosis pilaris, and plantar keratoderma.

**Figure 2:**
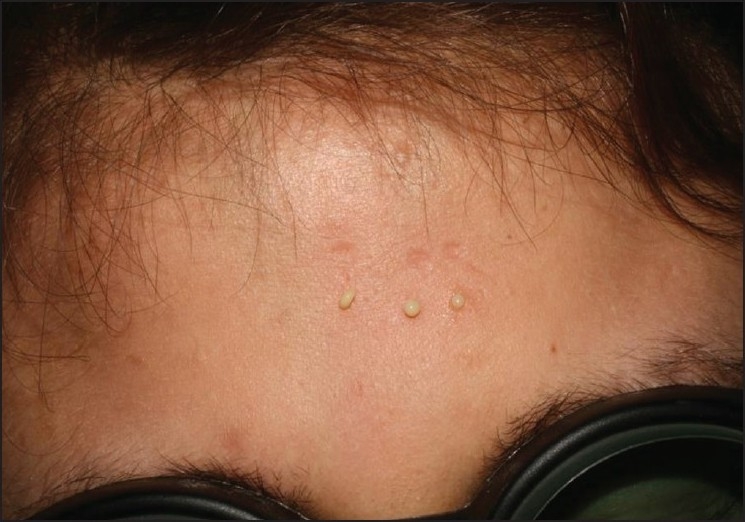
Oily and creamy material drained from Er: Yag laser created punctum

We treated the SM lesions with a 400 mJoule/pulse, 50.9 J/cm[2] of a Er: Yag laser, 1-mm spot size, without local anesthesia on the axilla, legs, and face, and 500 m Joule/pulse, 63.6 J/cm[2] on the anterior chest. Oily and creamy material drained out from the Er: Yag laser-created punctum [Figure 2]. A topical tetracycline ointment was used after the laser treatment. There were no secondary bacterial infections and the areas healed without scarring. No recurrence was observed during the following three months.

Numerous options are reported in the literature for the treatment of SM. Surgery is the most effective treatment, but may not be desirable for cosmetic reasons when multiple lesions are present.[2] There are many alternatives to surgery, such as follow-up without treatment,[2] CO_2_ laser therapy,[3] oral isotretinoin,[4] and cryotherapy.[5]

Although numerous SM treatment options exist, we conclude that cosmetic considerations limit the desirability of these options. Er: Yag laser treatment was well tolerated by the patient and no complications developed during or after the procedure. The cosmetic results were satisfactory. We suggest that Er: Yag laser might be a good treatment option for SM lesions.
